# Special Issue: Molecular Research and Insights into COVID-19: 2nd Edition

**DOI:** 10.3390/ijms262110614

**Published:** 2025-10-31

**Authors:** Manuela Rizzi, Pier Paolo Sainaghi

**Affiliations:** 1 Department of Health Sciences (DiSS), Università del Piemonte Orientale (UPO), 28100 Novara, Italy; 2 IRCAD (Interdisciplinary Research Center of Autoimmune Diseases), Università del Piemonte Orientale (UPO), 28100 Novara, Italy; 3 Department of Translational Medicine (DiMeT), Università del Piemonte Orientale (UPO), 28100 Novara, Italy; 4 Department of Internal Medicine and Rheumatology, Azienda Ospedaliero-Universitaria “Maggiore della Carità” di Novara, 28100 Novara, Italy; 5 CAAD (Center for Translational Research on Allergic and Autoimmune Diseases), Università del Piemonte Orientale (UPO), 28100 Novara, Italy

In December 2019, an outbreak of pneumonia of unknown origin in Wuhan (China) marked the beginning of coronavirus disease (COVID-19) pandemic. The public health emergency of international concern status ended in mid-2023 [1] but, at the time of writing, severe acute respiratory syndrome coronavirus 2 (SARS-CoV-2), the etiological agent of COVID-19, is still circulating worldwide, although the currently predominating variants are largely different from the original virus [2,3]. The ability of SARS-CoV-2 to constantly evolve toward new variants with increased fitness [4] is the basis of its conversion from the causative agent of the deadliest pandemic since Spanish flu to an endemic virus [5].

Since the beginning, COVID-19 has been described as a very heterogeneous disease, accounting for nearly asymptomatic as well as life-threating manifestations. Clinically symptomatic disease is recognized as a complex condition that, in the most critical patients, could evolve in interstitial pneumonia and acute respiratory distress syndrome associated with widespread severe inflammation (cytokine storm), dysregulated immune responses, impaired coagulation and, finally, severe multiorgan failure [6,7,8].

Since the beginning of the COVID-19 emergency, researchers’ knowledge about the disease pathophysiology has continuously grown. Although great progresses have been made in understanding the pathogenic mechanism and host responses, it is noteworthy that, in recent years, evidence of a new condition, characterized by chronic symptoms lasting for a long time after disease onset, has been accumulating. This condition has been defined as “long COVID” or “post-acute COVID” and represents a clinical challenge, as it is characterized by heterogeneous symptoms, affecting different clinical domains (i.e., muscular weakness and fatigue, cognitive impairment) [9,10,11].

To date, it is commonly well-accepted that both COVID-19 and long COVID share a critical hallmark, represented by the dysregulated immune response finally leading to the development of a vicious circle in which inflammation leads to tissue damage and impaired regeneration [12,13]. Nevertheless, some areas of uncertainty remain to be elucidated, thus encouraging new studies focused on both conditions, with the aim of identifying novel possible therapeutic targets.

From the therapeutic point of view, despite the significant efforts made by pharmaceutical research during the emergency through the development of novel pharmacological agents and the repurposing of already existing ones, only a limited number of specific and effective solutions have been marketed [14]. Moreover, most of the available therapeutic interventions, being targeted to limit viral replication, show some important drawbacks, represented by the need to be administered early during the disease course to be effective (i.e., molnupiravir, nirmartrelvir + ritonavir) and, in some cases, the need for in-hospital administration [15,16,17,18,19].

As a matter of fact, to date, the pharmacological treatment for COVID-19 relies on antivirals, immunomodulators and immunity-based therapies. Antivirals, by directly inhibiting viral entry and replication, appear to be effective in mild to moderate (molnupiravir and nirmatrelvir + ritonavir) as well as in severe (remdesivir) COVID-19, but need to be administered early in the disease course (ideally in the first few days of infection). Immunomodulators (i.e., corticosteroids like dexamethasone and cytokine inhibitors like tocilizumab), indeed, by contrasting the cytokine storm generated by SARS-CoV-2 infection, have been proven to be effective particularly in the case of severe disease manifestations. Finally, antibody-based therapies, such as monoclonal antibodies, by specifically neutralizing viral spike protein, have also been used to improve clinical outcomes, especially when administered early, even if these treatments are no longer used being non effective in neutralizing current SARS-CoV-2 variants [15,20].

Long COVID, with its characteristic plethora of symptoms of different nature, is emerging only in recent times as a clinical challenge. Considering that long COVID sequelae have been suggested to originate from an hyperinflammatory response due to SARS-CoV-2 persistent reservoir or from COVID-19-induced Epstein–Barr virus reactivation, it has been proposed that pharmacological compounds able to modulate cellular metabolism and/or autophagy mechanisms could be effective [21,22]. Due to the paucity of verified clinical trials dealing with this new condition, the available treatment recommendations are mainly based on expert’s opinions, thus fostering novel investigations aimed to optimize patients’ functions and to improve their quality of life [21,22].

To date, available evidence, mainly coming from observational studies and only in a limited part from controlled clinical trials, points out as promising preventive and/or therapeutic intervention in long COVID different agents, such as antivirals, metformin, naltrexone and immunomodulators such as dexamethasone and anakinra [23,24]. Furthermore, even in the context of long COVID, vaccines appear as a valuable intervention in preventing, at least partially, disease development, thanks to their effect in reducing persistent infection. As it has been observed that vaccine therapy in patients suffering of long COVID results in an immediate rapid increase in viral load, it is noteworthy that vaccination associated with antiviral therapy could represent a promising combined intervention to improve clinical outcomes in these patients [25].

An essential hallmark of pharmacological treatment in both conditions is represented by the great variability observed in patients’ response, along with the limited effectiveness of some of these interventions when administered in patients suffering of advanced disease and, above all, by the continuous emergence of novel viral variants, which can evade immune system response induced by currently available therapies. Although effective in the short term, these pharmaceutical interventions need continuous attention to ensure their constant update according to the evolution of viral variant prevalence and disease manifestations.

In this complex scenario, the increasing popularity of over-the-counter nutritional supplements used as add-ons to pharmacological therapy or as preventive agents is not surprising.

Considering that several active ingredients normally found in foods, such as vitamins and oligoelements, are known to positively affect immune system efficiency, during the early phases of the pandemic, several observational studies and clinical trials have investigated their potential supportive role. Among the most investigated over-the-counter solutions, great attention has been paid to lactoferrin [26,27], vitamin C [28,29,30], and omega 3 fatty acids [31,32], even if with inconclusive results.

Due to its well-recognized immunomodulatory action, vitamin D is the only nutritional supplement that has been studied in great detail by a number of research groups. Despite the large number of scientific reports dealing with its possible role in COVID-19 management, even for vitamin D, no clear therapeutic indications emerged due to the wide heterogeneity in the available evidence [33,34,35].

In addition to these classical nutritional supplements, remedies from Chinese and Indian medical traditions as well as herbal supplements and essential oils from folk traditions have been investigated, showing positive results as supportive therapies in patients with paucisymptomatic or mild disease manifestations [36,37].

Not surprisingly, the interest in complementary interventions is still high, also concerning long COVID. Also, in this context, evidence is accumulating about the role of vitamins and other dietary supplements as adjunctive treatments [24,38]. Due to the still-limited knowledge about long COVID etiology and evolution, while promising, these data still need to be supported by rigorous clinical trials before clear clinical guidelines could be defined.

Considering that dietary habits are modifiable risk factors for infection development, some studies focused also on plasma levels of selected food-derived compounds, showing how higher baseline levels of these elements (i.e., vitamin D, monolaurin, L-arginine) could exert a protective role, by ensuring a stronger immune response toward pathogen invasion [39,40,41,42,43,44].

Despite the conclusion of the pandemic era, it is worth nothing that SARS-CoV-2 is becoming an endemic virus, thus supporting the need for continuous attention towards new variants emergence to timely identify mutations and mitigate their impact on the global healthcare system. To reach this goal, a strict collaboration between national agencies worldwide is essential, as well as the definition of shared protocols to ensure high-quality data collection. Moreover, a tight monitoring of viral evolution and of disease manifestations is essential in order to ensure the constant update of the available therapeutic options, with the aim to develop tailored approaches based on the specific patient needs, to reduce adverse event risk and maximize the clinical outcome improvement.

Another important issue related to both COVID-19 and long COVID management is represented by the increasing interest towards non-pharmacological interventions. Although promising results have been obtained in selected study populations, it is essential to remember that nutritional supplements and traditional remedies cannot replace pharmacological interventions, but should be used only as adjuvants to improve drugs’ effectiveness and/or to reduce associated side effects. Considering the large availability of widespread reports, often lacking scientific validation, it is essential that the scientific community sustains an evidence-based information dissemination, in order to ensure patients’ safety and awareness regarding the currently available therapeutic options.

Considering this continuously evolving scenario, the aim of this Special Issue is to collect evidence about COVID-19 and long COVID pathophysiology, disease evolution and treatment, to offer a comprehensive overview of the most updated research in the field, thus contributing to an increase in healthcare professionals’ and policy-makers’ awareness of the challenges imposed by such a dynamic condition, where the attention should not only be focused on the original condition but also on its long-term sequelae.
